# The Bidirectional Correlation between Fundamental Motor Skill and Moderate-to-Vigorous Physical Activities: A Systematic Review and Meta-Analysis

**DOI:** 10.3390/children10091504

**Published:** 2023-09-04

**Authors:** Yu Liu, Zhen Li, Li Yuan, Zhixiong Zhou

**Affiliations:** 1 Institute for Sport Performance and Health Promotion, Capital University of Physical Education and Sports, Beijing 100191, China; liuyu2021@cupes.edu.cn (Y.L.); yuanli2019@cupes.edu.cn (L.Y.); 2 School of Physical Education and Sport Science, Fujian Normal University, Fuzhou 350117, China; qbx20220036@yjs.fjnu.edu.cn

**Keywords:** functional motor skills, moderate-to-vigorous physical activity, preschool children, meta-analysis

## Abstract

Background: Physical activity in early life has positive health effects, but few children meet the physical activity recommendations. Fundamental motor skills (FMS) are related to physical activity and according to the theory, physical activity drives them in the early years and vice versa. However, no study has conducted a meta-analysis of the association between moderate-to-vigorous intensity physical activity (MVPA) and early FMS. This meta-analysis examined the bidirectional correlation between MVPA and domain-specific FMS in preschool children. Methods: We searched PubMed, Embase, and Cochrane Library databases for articles published up to August 2023. Cross-sectional and longitudinal studies were included if they targeted children (3–6 years old) as the study population. The association between objectively measured MVPA and FMS was evaluated. Results: We found 445 titles and abstracts. A total of ten studies (eleven datasets) and 2514 children met the inclusion criteria, including eight cross-sectional studies and three longitudinal studies. When using MVPA as the exposure, no associations were found with locomotor skills (β = 0.83, 95% CI: −0.08, 1.74, *p* = 0.07) and gross motor skills (β = 2.72, 95% CI: −0.28, 5.72, *p* = 0.08), but an association with object management skills was found (β = 0.18, 95% CI: 0.06, 0.30, *p* = 0.001). When MVPA was used as the outcome, no associations were observed between locomotor skills (β = 0.06, 95% CI: −0.35, 0.47, *p* = 0.79), but associations with object management skills (β = 0.15, 95% CI: 0.02, 0.27, *p* = 0.02) and gross motor skill were found (β = 0.56, 95% CI: 0.38, 0.75, *p* = 0.001). The sensitivity analysis showed that the results must be treated with caution. Conclusion: We found that gross motor skill (exposure) was positively associated with MVPA (outcome) in preschoolers. Object management skills were positively associated with MVPA (exposure) and MVPA (outcome) in preschoolers. In contrast, MVPA as an exposure was not associated with locomotor skills and gross motor skills. The results may suggest that promoting FMS is important for preschool children’s MVPA.

## 1. Introduction

Physical activity plays a very important role in the development of children, and is related to their physical, mental, and cognitive development [1,2]. In childhood, physical activity can promote obesity, skeletal development and cognitive development, and psychosocial and cardiovascular development [3]. Lifetime physical activity habits (sporty and sedentary) often occur in childhood and continue over time [4], but weaken with age [5]. Physical labor has become an increasingly severe public health issue, causing weight gain in childhood [6,7,8], and bringing heavy economic pressure to society [9]. According to Tremblay et al.’s survey, children’s overall physical activity scores are very low/poor worldwide [10]. The rate of adherence to sports guidelines varies around the world; the results showed that 93% of children in Australia met the recommended standards, while only 62% of children in Canada underwent physical activity testing with an accelerometer [11,12]. A survey on the level of physical activity among preschool children shows that 27% to 100% of children meet the recommended standards [13]. Preschool children are at an important stage for physical activity and play an important role in physical development [1,2,3,14]. Due to the lifelong consequences of not participating in physical activity, it is necessary to provide more information to encourage teenagers to actively participate in physical activity [15].

Therefore, understanding the mechanism of body movement is very important. Fundamental motor skills (FMS) are important factors that affects people’s physical fitness. In the early stages, FMS opened a window of opportunity for children’s development, which included the following contents: stability (such as balance), movement (such as hopping and jumping), and object control. Handling techniques (such as grasping and throwing) [16]. Locomotor skills refer to the coordination between various movements of a person, including running, sprinting, jumping, hopping, horizontal jumping, and other skills. Object manipulation refers to the manipulation of other objects (such as clubs or balls), including throwing, receiving, kicking, rolling, hitting, dribbling, and other actions. Equilibrium is also confirmed as a measurement indicator of FMS [17]. Although it is widely regarded as the foundation of more complex and professional sports and physical activity [16,18,19,20], it is indispensable for the development of physical activity [21], indicating the mutual influence between the two [22]. Due to the development of movement styles, early childhood is an important period for developing this skill, and by preschool age, young children will begin to participate in games and activities that require them to apply this skill [23]. In fact, people believe that there is a mutually reinforcing dynamic connection between motor skills and physical activity [24,25], and this connection continues to strengthen from childhood to adolescence. In 2008, Stodden et al. proposed the concept of the interaction between FMS and physical activity [24]. They also pointed out that the level of intimacy between parents and children may vary during their child’s growth. Stodden et al. believe that physical activity during childhood is crucial for the development of FMS, which in turn affects later physical activity [24]. In addition, as children’s growth and their motor skills improve, their FMS level becomes increasingly important for their participation in physical activities [24]. If physical activity can be linked to FMS during childhood, and by observing how this connection changes, this hypothesis can be validated.

A meta-analysis has great application value in this area, with the aim of making research in this area more statistically significant. This study will contribute to improving the level of early childhood health and provide a scientific basis for decision making in early childhood health education and related departments. There have been literature reports on the interaction between FMS and self-reported body movements [26], but there is no meta-analysis exploring the interaction between body movements and preschool FMS. Given that physical activity and FMS can improve the physical condition of adults [27,28,29,30], a deeper understanding of the intrinsic relationship between these factors has become a focus of early public health research. To this end, we plan to conduct a systematic study and combine it with meta-analysis to explore the impact of moderate-to-vigorous-intensity physical activity (MVPA) on FMS.

## 2. Methods

This systematic review and meta-analysis was performed according to the PRISMA guidelines [31]. This systematic review is registered in the International Prospective Register for Systematic Reviews (PROSPERO), registration number 2022: CRD42022384345.

### 2.1. Literature Search

**Figure 1:**
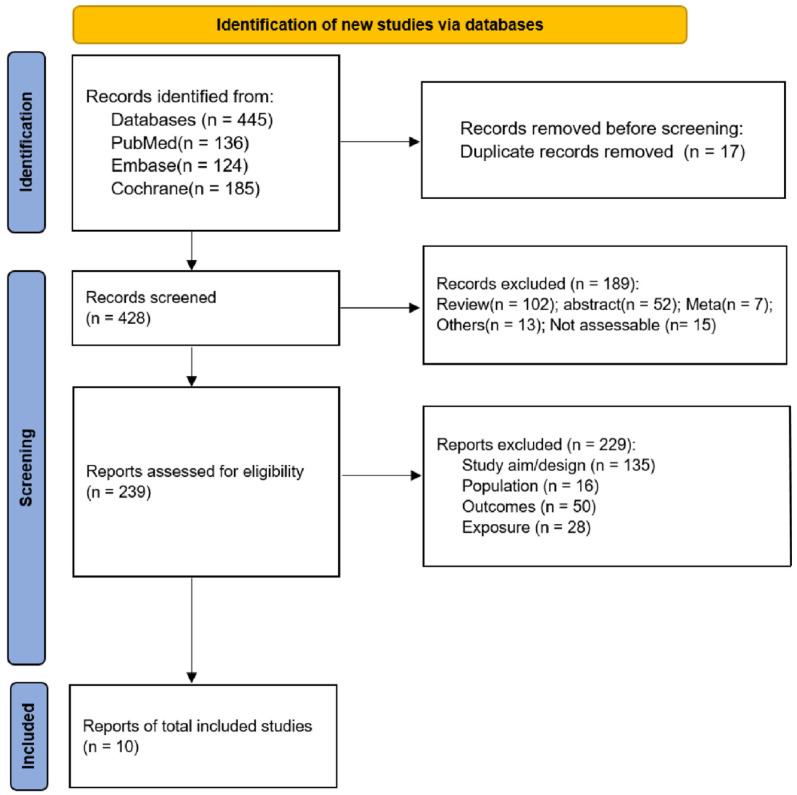
Flow diagram of the study selection process.

The applied strategy was based on the PICO principle [32]. A systematic literature search was conducted using the electronic bibliographic databases PubMed, Web of Science Core Collection, MEDLINE and Google Scholar with no date restrictions up to August 2023. Keywords were collected through experts’ opinions, literature review and controlled vocabulary (e.g., Medical Subject Headings (MeSH)). The search was limited to peer-reviewed, randomized controlled studies written in English. A Boolean search syntax was applied using the operators “AND”, “OR” and “NOT”. The following syntax is an example of a PubMed search: (“preschool children” OR “toddlers” OR “young children” OR “preschoolers”) AND (“moderate to vigorous physical activity” OR “MVPA” OR “physical exercise” OR “physical activity”) AND (“basic motor skills” OR “fundamental motor skills” OR “motor development” OR “motor abilities”). Search results were screened by two authors (ZL, YL). First, titles of all relevant articles were screened. Thereafter, abstracts and finally full texts were examined to confirm the inclusion. Reference lists of eligible articles were manually searched to further identify potentially relevant publications. If a study did not fulfill all criteria, the respective exclusion criterion was documented and the study was not considered for further analysis. In the case of disagreement between the two authors, a third coauthor (ZX) was consulted. An overview of the screening process is outlined in Figure 1.

### 2.2. Criteria for Study Inclusion and Exclusion

Studies were deemed eligible for inclusion if they satisfied the following predefined criteria: (1) Children in the study were between 3 and 6 years of age at baseline. (2) The research involved typically developing children and the measurement of MVPA was performed objectively using an accelerometer. (3) The total or global FMS score was reported in a study evaluated using validated methods. (4) The study reported the relationship between FMS and physical activity. (5) Cross-sectional or longitudinal design was used in the study. (6) The language was limited to English. The analysis excluded studies that did not meet the following criteria: (1) If the study did not provide subgroup data for participants aged 6 years or older, or if the average age of participants at baseline or in a cross-sectional study was above 6 years old. (2) A group of children who were facing developmental delays or physical impairments. (3) Research types: experience reports, conference abstracts, review articles and reviews. (4) The same patient groups were repeated in the publications.

### 2.3. Study Selection and Data Extraction

According to the search strategy and inclusion and exclusion criteria, two researchers independently screened the literature, and a third party was invited to make a judgment in case of disagreement. Literature screening was performed by first reading the title and abstract, and after excluding the obviously irrelevant literature, a further full text was read to determine final inclusion. 

The extracted data included the following: research data (e.g., authors, year of publication and country); population (e.g., age, gender and number of participants); measurement tools (e.g., physical activity measurement, and FMS measurement); outcome measures (MVPA threshold and children’s MVPA engagement for minutes per day); and analysis (correlation values between FMS and physical activity: coefficient (standardized β) and standard error (SE)). They were separated by two different researchers according to a predefined protocol.

### 2.4. Quality of the Evidence

Ultimately, ten studies entered the analysis. Two authors independently assessed the quality of the included studies and their risk of bias according to the Newcastle–Ottawa Scale (NOS) for pooled studies [33], and the Office of Health Research and Quality (AHRQ) guidelines for cross-sectional studies [34,35]. Each study was assessed by NOS in three key domains: participant selection, intergroup comparison, and the outcomes pertaining to the groups under investigation. NOS scores ranged from 0 to 9, and studies with NOS scores > 6 were considered positive. The studies are evaluated by AHRQ based on (1) how well the study was conducted and its internal validity; (2) the possibility of random errors; (3) the study’s external or overall validity; (4) the execution of advertisements; (5) selecting the displayed result; (6) selection of test results; (7) educational design; (8) fairness of intervention; and (9) conflict of interest in education administration. Qualitative analysis was carried out by 2 independent observers; all disagreements were effectively resolved through thorough and collaborative discussions, ultimately leading to the successful attainment of a consensus.

### 2.5. Statistical Analysis

All analyzes were conducted utilizing STATA SE 17.0 (StataCorp, College Station, TX, USA). Regression coefficients from unadjusted and adjusted analyzes were combined using random-effects meta-analysis. The statistical heterogeneity of the studies was calculated using Cochran’s Q test and the *I*^2^ index. *I*^2^ > 50% and Q-test *p* < 0.10 indicated high heterogeneity. *p* values < 0.05 were considered statistically different. A random-effects model is used if the heterogeneity is significant, and a fixed-effects model is used when the heterogeneity is moderate. Potential publication bias was not assessed using funnel plots and Egger’s test, as each quantitative analysis included <10 studies, where funnel plots and Egger’s test could give misleading results [36,37]. We performed a sensitivity analysis to further assess the reliability of our results. A sensitivity analysis was performed to investigate the possible influence of each individual study on the overall results by removing one study from the pooled analysis each time.

## 3. Results

### 3.1. Study Selection

Figure 1 illustrates the systematic study selection process. Initially, a comprehensive search identified a total of 445 records, from which 428 were screened after removing duplicates. Among them, 189 records were subsequently excluded based on predefined criteria. Subsequently, 239 full-text articles or abstracts were meticulously evaluated for eligibility, resulting in the exclusion of 129 studies. Ultimately, this study encompassed ten rigorous investigations (comprising nine datasets) and involved a cohort of 2514 children [22,38,39,40,41,42,43,44,45,46].

### 3.2. Study Characteristics

Table 1 provides a comprehensive summary of the ten studies incorporated in the review. These studies, published in English between 2009 and 2023, were conducted in five distinct countries: two studies were from the United States of America [38,39]; four were from Australia [40,43,44,45]; one was from Norway [22]; two were from the UK [41,46]; and one was from Finland [42]. In the cross-sectional studies, 1959 participants were included, while the longitudinal studies had 555 participants. The mean/median age of the children was 1.65–6.5 years, and the percentage of boys was 45–100% (Table 1). The study examined the impact of a wide variety of covariates (Table 1).

All included studies used accelerometers to measure MVPA. One dataset used an ActiGraph GT1M accelerometer, seven datasets used an ActiGraph GT3X+ accelerometer, one dataset used an ActiGraph GT4X+ accelerometer, one dataset used an ActiGraph 7164 uniaxial accelerometer, and one dataset used another accelerometer.

### 3.3. Quality Assessment

Study quality ranged from intermediate to high. According to the NOS, all three cohort datasets scored 7 points [22,38,39] (Supplementary Table S1a). Among the six cross-sectional datasets evaluated using the AHRQ tool, three scored 7 points [41,43,44], and five scored 8 points [38,40,42,45,46] (Supplementary Table S1b).

### 3.4. Locomotor Skills as the Outcome

**Figure 2:**
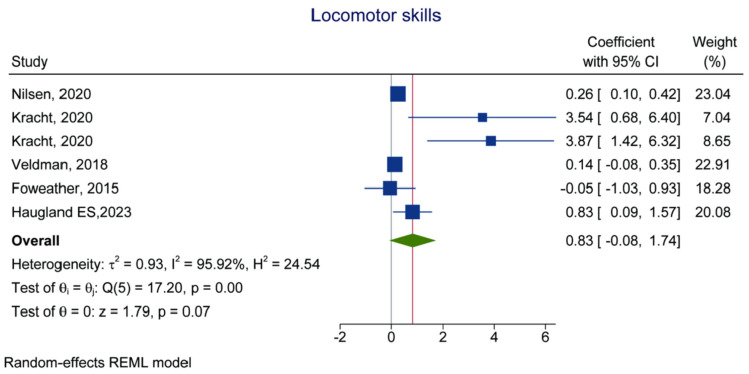
Forest plot of the association between MVPA (exposure) and locomotor skill (outcome) [22,38,44,41,45].

Six datasets examined the association between MVPA and locomotor skill [22,38,41,44,45]. MVPA and locomotor skills were not associated (β = 0.83, 95% CI: −0.08, 1.74, *p* = 0.07, *I*^2^ = 95.92%) (Figure 2 and Table 2).

### 3.5. Control Skills as the Outcome

**Figure 3:**
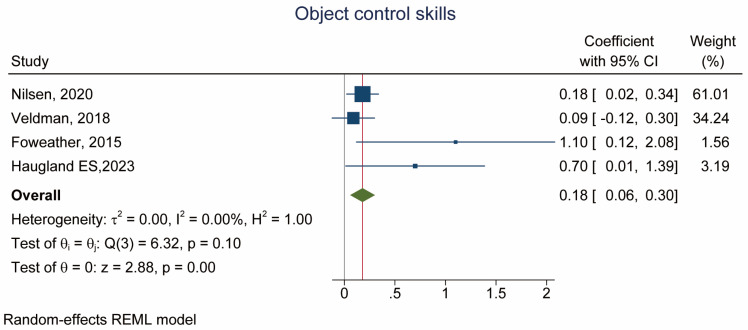
Forest plot of the association between MVPA (exposure) and object control skill (outcome) [22,44,41,45].

Four studies examined the association between MVPA and control skill [22,41,44,45]. There was a significant association between MVPA and control skills (β = 0.18, 95% CI: 0.06, 0.30, *p* = 0.001, *I*^2^ = 0.00%) (Figure 3 and Table 2).

### 3.6. Total Motor Skills as the Outcome

**Figure 4:**
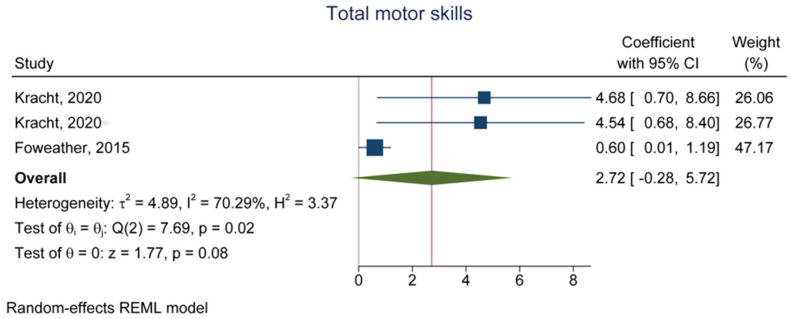
Forest plot of the association between MVPA (exposure) and total motor skill (outcome) [38,41].

Three datasets reported total motor skills [38,41]. The meta-analysis showed no association between MVPA and motor skills (β = 2.72, 95% CI: −0.28, 5.72, *p* = 0.08, *I*^2^ = 70.29%) (Figure 4 and Table 2).

### 3.7. MVPA as the Outcome

**Figure 5:**
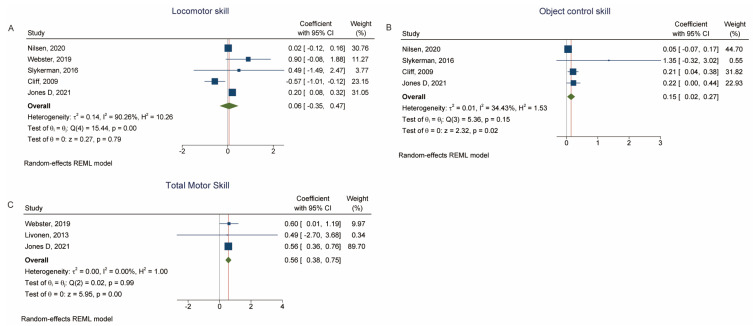
(**A**) Forest plot of the association between locomotor skill (exposure) and MVPA (outcome). (**B**) Forest plot of the association between object control skill (exposure) and MVPA (outcome). (**C**) Forest plot of the association between total motor skill score (exposure) and MVPA (outcome) [22,39,43,40,46,42].

Five studies examined MVPA as the outcome with locomotor skill as the exposure [22,39,40,43,46] and there was no association between MVPA and locomotor skill (β = 0.06, 95% CI: −0.35, 0.47, *p* = 0.79, *I*^2^ = 90.26%, P_heterogeneity_ = 0.001) (Figure 5A and Table 2). Associations were observed with object management skills [22,40,43,46] (β = 0.15, 95% CI: 0.02, 0.27, *p* = 0.02, P_heterogeneity_ = 0.15) (Figure 5B and Table 2) and gross motor skill [39,42,46] (β = 0.56, 95% CI: 0.38, 0.75, *p* = 0.001, *I*^2^ = 0.00%, P_heterogeneity_ = 0.99) (Figure 5C and Table 2).

### 3.8. Sensitivity Analyses

**Figure 6:**
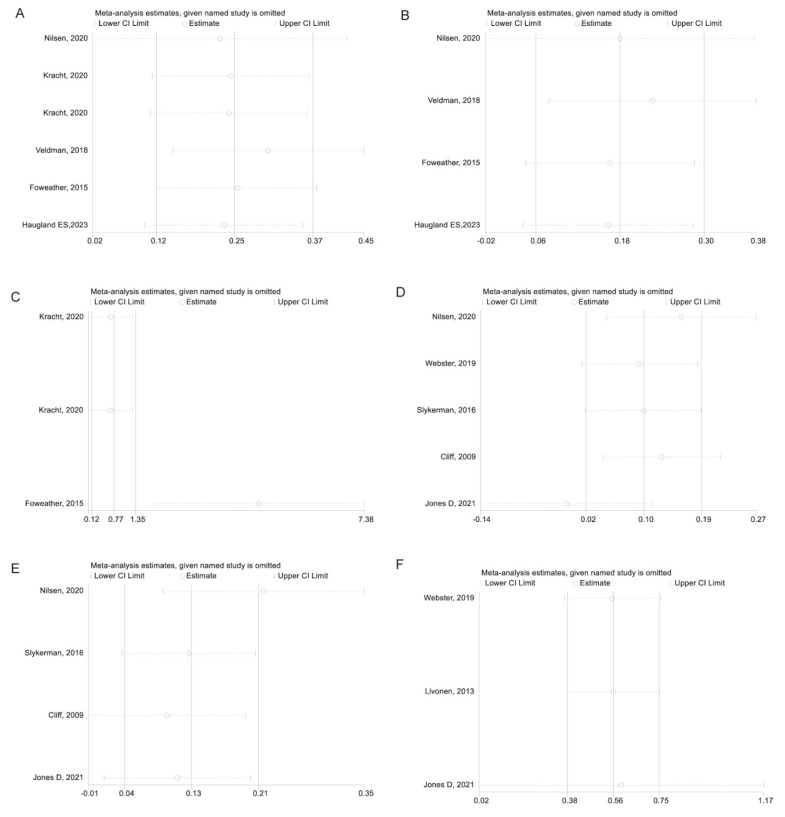
(**A**) Sensitivity analysis of the association between MVPA (exposure) and locomotor skill (outcome). (**B**) Sensitivity analysis of the association between MVPA (exposure) and object control skill (outcome). (**C**) Sensitivity analysis of the association between MVPA (exposure) and total motor skill (outcome). (**D**) Sensitivity analysis of the association between locomotor skill (exposure) and MVPA (outcome). (**E**) Sensitivity analysis of the association between object control skill (exposure) and MVPA (outcome). (**F**) Sensitivity analysis of the association between total motor skill (exposure) and MVPA (outcome) [22,38,44,41,45,39,43,40,46,42].

The sensitivity analysis for the association between MVPA (as exposure) and locomotor skill (as outcome) showed that sequentially excluding Foweather et al. [41] and dataset b from Kracht et al. [38] made the association turn significant (Figure 6A). Excluding Foweather et al. [41] also changed the results for MVPA as the exposure and control skill (Figure 6B) and motor skill (Figure 6C) as the outcomes. Figure 6D–F showed that all three analyses using MVPA as the outcome were robust.

## 4. Discussion

Previously, there was almost no meta-study on the binary association between physical activity and FMS in preschool children. So, the purpose of this meta-analysis is to detect the binary association between MVPA and FMS. The study found that there was a significant positive relationship between children’s technical scores (based on exposure value) and MVPA (based on performance value). There was no significant relationship between MVPA (contact) and factors such as locomotor skills and gross motor skills. Sensitivity analysis shows that research results should be handled with caution. To the researcher’s knowledge, it is the first study to conduct a bidirectional correlation between MVPA and FMS.

There is a mutual and dynamic connection between motor skills and physical activity [24,25]. The advantage of this experiment is that even if MVPA is used, MVPA and motor skills have been tested from two aspects. After using MVPA as a training effect, it was found that it has a significant correlation with the overall motor skill score, indicating that improving the overall motor skill will promote the training effect of MVPA. This study confirms the important role of FMS levels in the participation of physical activities in preschool children, and Stodden et al. [24]’s conceptual model and previous studies [26,47,48,49,50] confirm this hypothesis. However, Nilsen et al. [22] did not show any effect of FMS on body movement at baseline. However, due to different ages and covariates, direct experimental comparisons cannot be made. The development level and speed of FMS in early childhood may vary to varying degrees [47,51,52], and at a certain age, the correlation between FMS is more pronounced than in other populations. Because it includes a large number of experiments, this meta-analysis can provide an explanation for this.

However, this meta-analysis did not find the effect of MVPA on FMS but found an association with object management skills. It might be the case that MVPA often involves the use of objects or equipment and requires coordination and organization. Many forms of MVPA involve the manipulation or interaction with objects or equipment. Children’s hand–eye coordination, fine motor control, and object control skills play a role in performing these activities effectively and efficiently. Developing good object management skills can improve coordination, precision, and efficiency in physical activities that involve objects or equipment, ultimately enhancing the overall experience and effectiveness of MVPA. During the contact process, there is no significant correlation between the dissolution of MVPA and the constituent elements of motor ability, indicating that the practice of MVPA is not helpful for young children’s motor ability. However, not all RCTs have confirmed this [53,54,55]. The key is that sensitivity analysis shows that MVPA cannot be used for contact analysis, and the cross-sectional data sets of Foweather et al. [41] and Kracht et al. [38] make the correlation more pronounced. Therefore, the current meta-analysis cannot prove a conflict with the hypothesis proposed by Stodden et al. [24]. It is also emphasized that in-depth investigation is needed on this topic. This can also be attributed to the delayed effect of physical activity on FMS. In fact, Nilsen et al. [22] found that FMS improved two years later. This lag effect was not taken into account in this experiment. In addition, the correlation between FMS and physical activity is more pronounced at a certain age stage.

Because childhood is an important stage for developing healthy behaviors, including physical activity [56], developing motor skills will lay a good physical activity habit for future life [4]. Currently, children’s motor abilities are very weak [57,57], and it is necessary and necessary to first create an environment conducive to their motor abilities [53,56,58].

The reviewed longitudinal studies do not offer sufficient evidence to either support or disprove Stodden et al.’s hypothesis that physical activity contributes to the development of FMS in early childhood [24]. To ensure the effectiveness of conceptual models, it is crucial to conduct additional research on the correlation between physical activity and FMS. The data collection process should start in early childhood and continue at regular intervals until adulthood, including middle to late childhood and adolescence. By doing so, we can strengthen the evidence and determine the direction of the association, which will enable the development of appropriate interventions based on age and skill level. Another shortage is the limited number of studies on FMS for each category of physical ability, which has resulted in a small sample size and low statistical power [59]. As a consequence, the results may be more uncertain and have limited ability to accurately estimate the overall effect. It is therefore recommended that future research should prioritize addressing this issue to improve the accuracy of the findings.

The systematic review of this study has the advantages of rigorous retrieval, screening, and meta-analysis. However, we must recognize the limitations of our review power. FMS is complex, and the concepts and measurement methods of FMS vary among researchers, making comparisons between various studies more complex. In addition, there is no “gold standard” for evaluating children’s FMS. This article mainly uses an experimental battery based on TGMD, which has been widely accepted by children in kindergartens. It should be pointed out that TGMD is developed by the United States of America and includes some projects that have little or no cultural relationship with other countries (such as baseball bat and rebound). In addition, the sample size of this survey is relatively small. Most of the experiments are horizontal. Tracking research helps understand the relationship between the development of basic motor skills and physical movements in children’s growth process. Both groups of patients’ MVPA were evaluated using acceleration sensors. The latest research has found that accelerometers are not the “gold standard” of MVPA, and there are serious deviations in the testing of subject projection techniques [60]. Therefore, it is possible that we underestimated the correlation of FMS with MVPA. Finally, caution must be taken when interpreting the results of this meta-analysis, as individual studies influenced the sensitivity analyzes of FMS outcome.

## 5. Conclusions

In summary, the total skill score (exposure) was positively associated with MVPA (outcome) in preschoolers. In contrast, MVPA as an exposure was not associated with total skill scores and locomotor skills. The results may suggest that promoting FMS is important for preschool children’s MVPA.

## Figures and Tables

**Table 1:** Characteristics of the included studies.

Study	Design	Country	Sample Size			Age (Year, Mean or Median)			MVPA (min/day)			Physical Activity	Fundamental Motor Skills	Cut-Off Value of MVPA	Covariates
			Total	Boys	Girls	Total	Boys	Girls	Total	Boys	Girls				
Jones et al., 2021 [46]	Cross-sectional study	UK	325	170	155	5.5 (0.3)	/	/	100.1 (23.4)	/	/	Accelerometer	TGMD-2	≥1680 cpm	Sex, age at exposure, BMI-z and SES
Haugland et al., 2023 [45]	Cross-sectional study	Australia	952	488	464	4.3 (0.9)	4.3 (0.9)	4.4 (0.9)	75 (17)	79 (17)	70 (15)	ActiGraph GT3X+	TGMD-3	≥2296 cpm	Sex, age, BMI, SES, accelerometer wear time, and assessor for FMS outcomes
Kracht et al., 2020 [38]	Prospective cohort study	USA	53	28	25	3.2 ± 0.5	/	/	102 (30)	/	/	ActiGraph GT4X+ accelerometer	TGMD-3	≥420 counts/15 s	Baseline raw score (locomotor, ball skills, or TGMD-3), race, the other behaviors, and accelerometer wear time
Nilsen et al., 2020 [22]	Prospective cohort study	Norway	376	376	0	4.7 ± 0.9	/	/	70 (14)	/	/	ActiGraph GT3X+ accelerometer	TGMD-3	≥2296 cpm	Sex, baseline age, baseline BMI, parental education and income level, accelerometer wear time at both time points, and the person scoring FMS at both time points
Webster et al., 2019 [39]	Prospective cohort study	USA	126	58	68	3.4 ± 0.5	3.4 ± 0.5	3.3 ± 0.5	/	120 (36)	90 (30)	ActiGraph GT3X+ accelerometer	TGMD-3	≥1680 cpm	Age, sex, household, income, acceleromotor wear time
Cliff et al., 2009 [40]	Cross-sectional study	Australia	46	25	21	/	4.24 ± 0.69	4.35 ± 0.64	/	23.25 (32.4)	22.67 (15.37)	ActiGraph 7164 uniaxial accelerometers	TGMD-2	MVPA, >2460, >3248 and, >3564 cpm, for 3-, 4-, and 5-year-olds	Age, SES, BMI
Foweather et al., 2015 [41]	Cross-sectional study	UK	99	52	47	4.6 ± 0.5	4.7 ± 0.6	4.6 ± 0.5	/	92.8 (30.7)	76.7 (26.7)	ActiGraph GT1M accelerometer	TGMD-2	≥1680 cpm	Clustering, age, sex, standardized BMI, accelerometer wear time
Kracht et al., 2020 [38]	Cross-sectional study	USA	107	48	59	3.4 ± 0.6	/	/	102 (36)	/	/	ActiGraph GT3X+ accelerometer	TGMD-3	≥420 counts/15 s	Age, sex, race, and other behaviors
Iivonen et al., 2013 [42]	Cross-sectional study	Finland	37	17	20	4.1 ± 0.34	4.2 ± 0.38	4.02 ± 0.29	/	61.50 (20.39)	59.90 (18.42)	ActiGraph GT3X accelerometer	APM Inventory manual and test booklet by Numminen (1995)	≥196 counts/5 s	Sex, age, BMI, total skill score
Slykerman et al., 2016 [43]	Cross-sectional study	Australia	109	59	50	6.5 ± 1.0	/	/	/	76.1 (20)	57.0 (17.7)	ActiGraph GT3X+ accelerometer	TGMD-2	≥2296 cpm	Age, sex, accelerometer wear time, English speaking background
Veldman et al., 2018 [44]	Cross-sectional study	Australia	284	151	133	1.65 ± 0.35	1.65 ± 0.35	1.64 ± 0.34	/	528.35 ± 124.32	547.18 ± 100.28	ActiGraph GT3X+ accelerometer	PDMS-2	>1680 cpm	Sex, age, BMI.

TGMD-2, Test of Gross Motor Development, 2nd Edition; PDSM-2, Peabody developmental motor scales second edition; TGMD-3, Test of Gross Motor Development, 3rd edition; SES, socioeconomic status; BMI, body mass index; MVPA, moderate-to-vigorous physical activity; cpm, counts per minute.

**Table 2:** Bidirectional association between MVPA and FMS.

	N	Standardized β (95% CI)	*p*	*I*^2^ (%)	P_heterogeneity_
Fundamental motor skills (outcome)					
Total	3	2.72 (−0.28, 5.72)	0.08	70.29	0.02
Locomotor skills	6	0.83 (−0.08, 1.74)	0.07	95.92	0.001
Object control skills	4	0.18 (0.06, 0.30)	0.001	0.00	0.10
Moderate-to-vigorous physical activity (outcome)					
Total	3	0.56 (0.38, 0.75)	0.001	0.00	0.99
Locomotor skills	5	0.06 (−0.35, 0.47)	0.79	90.26	0.001
Object control skills	4	0.15 (0.02, 0.27)	0.02	34.43	0.15

CI: confidence interval.

## Data Availability

All data generated or analyzed during this study are included in this published article and its supplementary information files.

## Supplementary Materials

The following supporting information can be downloaded at https://www.mdpi.com/article/10.3390/children10091504/s1. Table S1a. Evaluation of the cohort datasets using the NOS; Table S1b. Evaluation of the cross-sectional datasets using the AHRQ tool.
