# Supplementary material for: The Bidirectional Correlation between Fundamental Motor Skill and Moderate-to-Vigorous Physical Activities: A Systematic Review and Meta-Analysis
Source: Children (Basel). 2023 Sep 4;10(9):1504. doi: 10.3390/children10091504 (PMC10527642; doi:10.3390/children10091504)
Supplement: Supplementary file 1 [file children-10-01504-s001.zip › children-2484907-supplementary.pdf]

Supplementary Table S1a. Evaluation of the cohort datasets using the NOS

Study	Representativeness of the exposed cohort	Selection of the non-exposed cohort	Ascertainment of outcome	Demonstration that outcome of interest was not present at the start of the study	Comparability of cohorts based on the design or analysis	Assessment of outcome	Was follow-up long enough for outcomes to occur	Adequacy of follow up of cohorts	Total quality scores
Kracht, 2020 [38]	☆	☆	☆	/	☆☆	☆	/	☆	7
Nilsen, 2020 [22]	☆	☆	☆	/	☆☆	☆	☆	/	7
Webster, 2019 [39]	☆	☆	☆	/	☆☆	☆	/	☆	7

Supplementary Table S1b. Evaluation of the cross-sectional datasets using the AHRQ tool

**Study**	**Define the source of information**	**List inclusion and exclusion criteria for exposed and unexposed subjects or refer to previous publications**	**Indicate time period used for identifying patients**	**Indicate whether or not subjects were consecutive if not population-based**	**Indicate if evaluators of subjective components of the study were masked to other aspects of the status of the participants**	**Describe any assessments undertaken for quality assurance purposes**	**Explain any patient exclusions from the analysis**	**Describe how confounding was assessed and/or controlled**	**If applicable, explain how missing data were handled in the analysis**	**Summarize patient response rates and completeness of data collection**	**Clarify what follow-up, if any, was expected and the percentage of patients for which incomplete data or follow-up was obtained**	**Total quality scores**
Cliff, 2009 [40]	☆	☆	☆	☆	☆	/	/	☆	/	☆	☆	8
Foweather, 2015 [41]	☆	/	☆	☆	☆	/	/	☆	/	☆	☆	7
Kracht, 2020 [38]	☆	☆	☆	☆	☆	/	/	☆	/	☆	☆	8
Iivonen, 2013 [42]	☆	☆	☆	☆	☆	/	/	☆	/	☆	☆	8
Slykerman, 2016 [43]	☆	/	☆	☆	☆	/	/	☆	/	☆	☆	7
Veldman, 2018 [44]	☆	/	☆	☆	☆	/	/	☆	/	☆	☆	7
Jones D,2021 [46]	☆	☆	☆	☆	☆	/	/	☆	/	☆	☆	8
Haugland ES,2023 [45]	☆	☆	☆	☆	☆	/	/	☆	/	☆	☆	8
